# Personalized whole-body modeling links gut microbiota to metabolic perturbations in Alzheimer’s disease

**DOI:** 10.1080/19490976.2026.2732659

**Published:** 2026-09-28

**Authors:** Tim Hensen, Lora Khatib, Lucas Patel, Daniel McDonald, Antonio González, Siamak MahmoudianDehkordi, Colette Blach, Rob Knight, Rima Kaddurah-Daouk, Ines Thiele

**Affiliations:** a Digital Metabolic Twin Centre, University of Galway, Galway, Ireland; b School of Medicine, University of Galway, Galway, Ireland; c Department of Paediatrics, University of California San Diego, La Jolla, CA, USA; d Neurosciences Graduate Program, University of California San Diego, La Jolla, CA, USA; e Bioinformatics and Systems Biology Program, University of California San Diego, La Jolla, CA, USA; f Medical Scientist Training Program, University of California, San Diego, La Jolla, CA, USA; g Department of Psychiatry and Behavioural Sciences, Duke University, Durham, NC, USA; h Centre for Microbiome Innovation, University of California, San Diego, La Jolla, CA, USA; i Department of Computer Science and Engineering, University of California, San Diego, La Jolla, CA, USA; j Shu Chien-Gene Lay Department of Bioengineering, University of California, San Diego, La Jolla, CA, USA; k Halıcıoğlu Data Science Institute, University of California, San Diego, La Jolla, CA, USA; l Hong Kong University of Science and Technology Jockey Club Institute for Advanced Study, Hong Kong University of Science and Technology, Hong Kong SAR, China; m Duke Institute of Brain Sciences, Duke University, Durham, NC, USA; n Department of Medicine, Duke University, Durham, NC, USA; o School of Microbiology, University of Galway, Galway, Ireland; p Ryan Institute, University of Galway, Galway, Ireland; q APC Microbiome Ireland, Cork, Ireland

**Keywords:** Gut microbiome, constraint-based modeling, host–microbiome co-metabolism, metagenomics, metabolomics, Alzheimer’s disease

## Abstract

The human gut microbiome has been linked to metabolic disturbances in Alzheimer’s disease (AD). However, the mechanisms by which gut microbes might influence metabolic dysfunction in AD remain poorly understood. Previously, gut microbiome-personalized whole-body models of human metabolism have been applied to predict how altered gut microbiome compositions may influence metabolites in the blood of healthy aging individuals with increased risk of AD. However, these previous results have not been validated in AD. In this study, we aimed to test these prior predictions in a cohort of AD dementia patients and individuals with mild cognitive impairment (MCI) and a probable AD diagnosis. Therefore, we created gut microbiome-personalized whole-body metabolic models for 34 AD dementia patients, 51 MCI patients, and 298 healthy controls. These *in silico* models were profiled to predict the metabolic influences of gut microbiomes on blood metabolites with previously reported alterations in AD. We found increased capacities of the *in silico* host-microbiome co-metabolism to produce S-adenosyl-L-methionine, L-arginine, creatine, taurine, and formate in the blood of AD patients. The metabolic predictions were then linked to key microbial taxa using a novel method that combines modeling-informed prediction sensitivity to alternative microbial abundances with LASSO-based taxonomic stability selection and elastic net regressions. This method found that increased relative abundances of *Bacteroides uniformis* and *Bacteroides thetaiotamicron* in AD were major factors driving the predicted metabolic changes. Furthermore, the metabolic predictions were associated with allelic variations in the *APOE* risk gene in healthy individuals, confirming our previous findings. In conclusion, we identified blood metabolites with known links to AD that were differentially influenced by gut microbiota in AD, and identified possible microbial drivers of these predicted shifts in host-microbiome interactions. These findings may facilitate the development of microbiome-informed treatments of AD.

## 1. Introduction

Alzheimer’s disease (AD) is the most common neurodegenerative disease. AD is generally characterized by a progressive loss of neurons, extracellular accumulation of amyloid beta proteins, and intracellular accumulation of hyperphosphorylated tau proteins.^1^ ^,^ ^2^ In addition, several major risk factors for AD have been identified, such as old age, lower cognitive functioning, and being a carrier of the *APOE* ε 4 allele.^3^ However, the mechanisms linking these risk factors to AD remain poorly understood. Emerging evidence has shown that microbiota inhabiting the gastrointestinal tract may influence AD pathology by acting on the gut-brain axis, which comprises bidirectional communication pathways connecting gut microbes to the brain.^4^ Previous reports have linked several microbial metabolites to AD, such as secondary bile acids,^5^ short-chain fatty acids,^6^ indoles,^7^ and trimethylamine N-oxide.^8^ However, the network-wide effects of microbial contributions to the gut-brain axis in AD remain largely unknown.

In addition to alterations in microbial metabolites, metabolomic studies have identified widespread metabolic perturbations in AD patients. A recent systematic review by Yin et al.^9^ identified 396 AD-associated metabolites across 66 pathways. Commonly found perturbed pathways in AD were arginine and proline metabolism, glutamate metabolism, phenylalanine metabolism, cysteine and methionine metabolism, and taurine and hypotaurine metabolism.^9^ To elicit the effects of gut microbiota on these perturbed pathways and identify the metabolic perturbations influenced by the gut microbiome, computational modeling methods have enabled the prediction of isolated gut microbiome influences on human metabolism,^10^ independent of other drivers of metabolism, such as disease progression, dietary habits, and environmental exposures.

*In silico* genome-scale metabolic models of gut microbiome communities have been a powerful tool for predicting the metabolic host-microbiome crosstalk in health and disease.^10^ ^,^ ^11^ These metabolic microbiome community models integrate metagenomic data with comprehensive microbial genome-scale metabolic reconstruction resources, such as AGORA2^12^ and APOLLO,^13^ and enable mechanistic investigations of personalized gut microbiome metabolism using constraint-based optimization methods, such as flux balance analysis (FBA).^14^ Microbiome community models have been previously used to predict altered metabolic features of gut microbiomes in inflammatory bowel diseases,^15^ ^,^ ^16^ colorectal cancer,^17^ and Parkinson’s disease.^18^ ^,^ ^19^ Microbiome community models, however, cannot model the bidirectional metabolic influences that the host and gut microbiome exert on each other, nor can they investigate microbial influences on human metabolites. To model host-microbiome crosstalk, recent studies have incorporated personalized microbiome community models into sex-specific and organ-resolved whole-body metabolic models (WBMs),^20^ creating integrated models of host-microbiome co-metabolism. These microbiome-personalized WBMs have been previously utilized to study host-microbiome co-metabolism in inflammatory bowel diseases^21^ and AD dementia.^22^ ^,^ ^23^

Previously, we have utilized gut microbiome-personalized WBMs to predict the contributions of gut microbiota to blood metabolites that may influence AD phenotype, and could associate these predictions with major risk factors of AD, including increasing age, *APOE* ε 4 carrier status, and global cognitive scores.^22^ This prior analysis associated the predicted gut microbiome influences on arginine metabolism, methionine metabolism, and secondary bile acid metabolism with an increased risk of developing AD, and identified *Eggerthella lenta* as a potential key contributor to the secondary bile acids, deoxycholate, and lithocholate. However, our previous study had important limitations. The predicted metabolic associations with the AD risk factors were made for healthy aging individuals and have not been validated in an independent cohort of AD patients. Secondly, poor correlations have been observed between the metabolic predictions and plasma metabolomics from the same samples. This lack of data agreement has been attributed to previously reported challenges that may arise when comparing human gut metagenomic and plasma metabolomic abundances, such as incomplete metagenomic and metabolomic annotations, temporal mismatches in sample collection, and differential interactions with factors, such as host genetics, dietary intake, environmental interactions, and disease progression.^24^ ^,^ ^25^ In addition, the use of lower-resolution 16S rRNA metagenomic data may have also reduced the modeling capacity to predict plasma abundances in corresponding samples. This previous study also identified potential microbial contributors to those metabolic predictions. However, the methods used in that study could not identify microbial contributors to non-microbial metabolites with microbe-derived precursors. Secondly, this prior study could not account for mechanistic constraints on microbial contributions to circulatory metabolites that may arise from model-specific diet–microbe–host interactions.

In this study, we followed up on our prior predictions by investigating these findings with an improved study design in an independent cohort that included individuals with mild cognitive impairment (MCI) and AD dementia patients. We utilized high-resolution shotgun metagenomics data and larger, updated microbial genome-scale reconstruction libraries, which enabled improved coverage of the sampled gut microbiome samples in the *in silico* models. We further introduced a novel mechanism-informed bioinformatic method for predicting microbial contributors to predicted blood metabolites in microbiome-personalized WBMs. A key feature of this bioinformatic method is that it enables the prediction of indirect microbial influences on distal non-microbial metabolites by utilizing the mechanistic structure of microbiome-personalized WBM.

Our analysis revealed that the gut metagenomes of MCI and AD dementia patients had increased capacities to influence circulatory metabolic levels of S-adenosyl-L-methionine, L-arginine, creatine, taurine, and formate, confirming and expanding our previous predictions. Crucially, these metabolites were not changed in the associated plasma metabolomic measurements. Our approach for identifying key microbial contributors to human metabolites determined that increased relative abundances of *Bacteroides uniformis* and *Bacteroides thetaiotaomicron* were a key factor for the AD-associated predicted fluxes in blood. Lastly, we demonstrated that the predicted circulatory metabolic predictions in MCI and AD dementia patients were also associated with *APOE* allele carrier status in healthy individuals. Taken together, this study identified shifts in gut microbial contributions to circulatory metabolites from healthy to MCI and AD dementia phenotypes, identified *B. uniformis* as a major contributor to these predictions, and showed that an *APOE* ε 4 carrier may be involved in preclinical host-microbiome crosstalk.

## 2. Results

We aimed to predict the metabolic influences of gut microbiota on a curated list of 38 blood metabolites in MCI and AD patients. To that end, we obtained gut metagenomic samples from a cohort of 383 American participants that included 298 healthy controls, 51 MCI patients with a presumptive AD diagnosis, and 34 AD dementia patients. The gut metagenomic samples were used to generate microbiome-personalized WBMs, which were given an average Western metabolic diet. FBA^14^ was then performed to interrogate the models on their maximal flux capacity to accumulate each metabolite in the WBM blood compartment (Figure 1; Methods). The predicted metabolic fluxes were associated with MCI and AD status to identify potential changes in metabolic contributions from gut microbiota. We then tested whether the *in silico* flux predictions could be correlated with plasma metabolite abundances in a subset of 168 individuals. Next, we identified the key microbial contributors to metabolites of interest by first performing feature selection to reduce the search space of possible microbe-metabolite associations and then determining the best associations between flux predictions and associated microbial species. Lastly, we validated our prior findings on AD risk markers by associating the AD-associated flux predictions with MoCA scores and *APOE* genotype status in a healthy subcohort.

**Figure 1.**
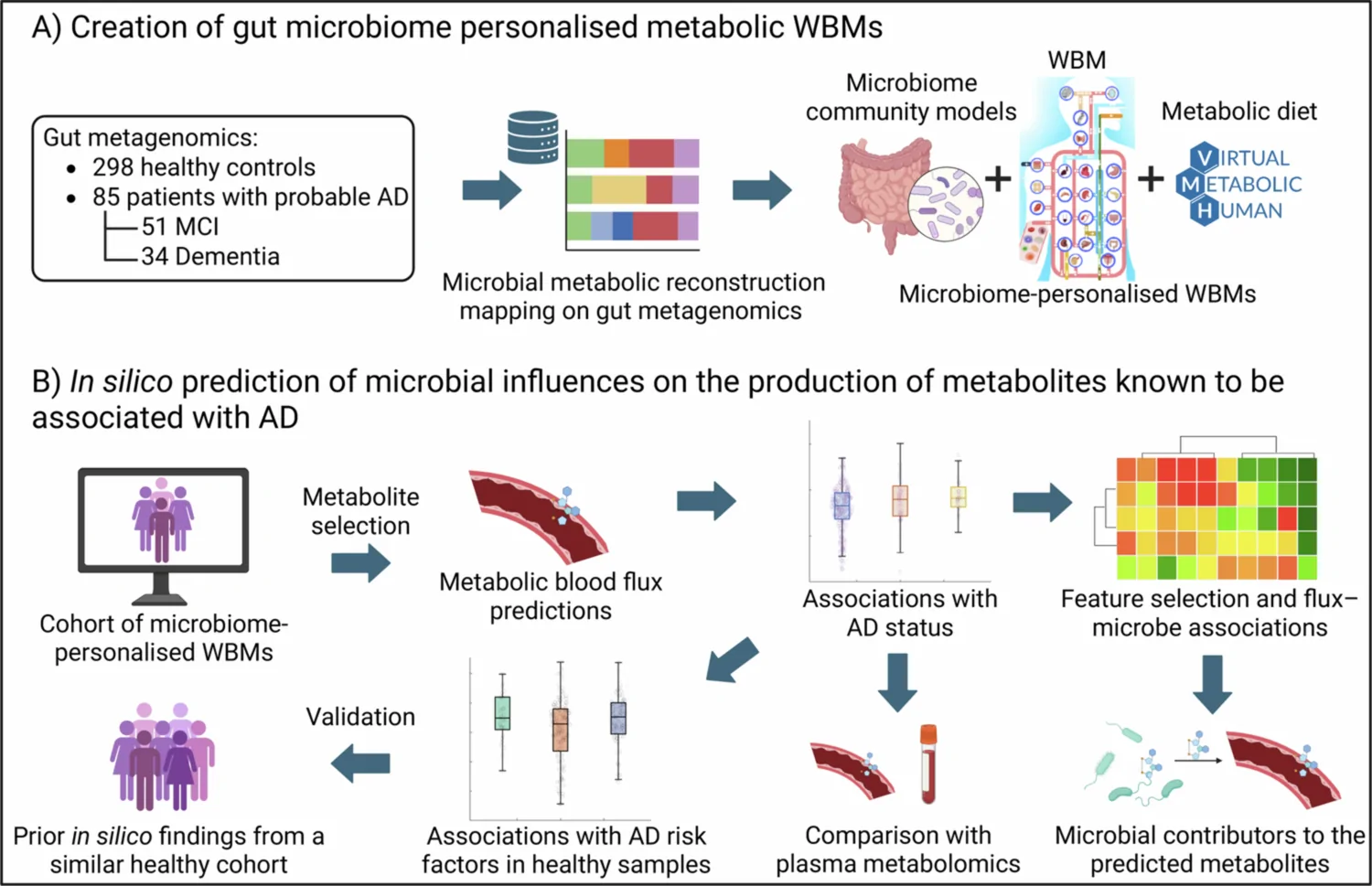
Schematic overview of the study design. (A) Gut metagenomics samples were available from 298 healthy controls and 85 patients with probable AD. Fifty-one of these patients had mild cognitive impairment (MCI), while the remaining 34 were diagnosed with dementia. The microbial species in the gut metagenomics data were then mapped onto corresponding microbial metabolic reconstructions in the AGORA2^12^ and APOLLO^13^ resources. These microbial reconstructions were then combined into microbiome community models and personalized with their corresponding relative abundances. The microbiome community models were then used to personalize standard male and female WBMs corresponding to the sex of each microbiome sample donor, and were given an average Western metabolic diet. (B) Metabolic blood fluxes, representing the capacity of the microbiome-personalized WBMs to accumulate a metabolite, were then predicted for 38 selected metabolites. These metabolites were selected as they have known associations with AD and were previously analyzed using microbiome-personalized WBMs to predict microbial associations with risk factors for AD dementia. The flux predictions were associated with MCI and AD dementia patients to find altered gut microbial influences on these metabolites in MCI and AD patients. The predicted metabolic associations were then compared with corresponding plasma metabolomic abundances from the same individuals. Next, the microbial contributors to the predicted fluxes were identified by applying a LASSO regression-based feature selection approach and then determining the best microbe-metabolite associations. Lastly, the predicted metabolites with altered flux predictions in MCI or AD dementia patients were associated with Montreal Cognitive Assessment (MoCA) scores and *APOE* genotype in a subset of healthy individuals to validate prior WBM modeling predictions. This figure was created in BioRender.

### 2.1. Study cohort

In this study, we used gut metagenomic data from a cross-sectional cohort of 400 participants that were sampled through the Alzheimer Gut Microbiome Project (Methods). The cohort included 308 cognitively healthy controls, 53 individuals with MCI and a presumptive diagnosis of AD, and 39 individuals with diagnosed AD dementia. However, before sample analysis, we removed two samples, which were collected much earlier than all other samples: one sample from a patient with schizophrenia and one sample from a participant who had a partial colon removal before sample collection, and thirteen samples that were outliers due to having a low read number, i.e., fewer than one million metagenomic reads, due to a species coverage in the AGORA2^12^ or APOLLO^13^ metabolic reconstruction resources below 25%. After removing these samples, 383 metagenomic samples remained available for analysis, including 298 healthy controls, 51 MCI patients with a presumptive diagnosis of AD, and 34 AD dementia patients (Table 1).

**Table 1.** Cohort characteristics of healthy controls, AD patients with mild cognitive impairment (MCI), and AD dementia patients.

Variable	*N*	Healthy controls (*N* = 298 )	MCI (*N* = 51 )	AD dementia (*N* = 34 )	*P*-value^†^
Female samples, *N* (%)	383	200 (67.11%)	25 (49.02%)	14 (41.18%)	1.33e-03
Age in years, mean (SD)	383	72.29 (7.4)	76.33 (7.45)	76.65 (6.69)	8.71e-05
Years of education, mean (SD)	383	16.44 (2.57)	16.57 (2.77)	15.85 (2.64)	3.83e-01
BMI in kg/m^2^, mean (SD)	347	27.39 (5.36)	27.68 (4.95)	24.63 (3.78)	1.78e-02
MoCA test score, mean (SD)	361	26.83 (2.5)	23.02 (3)	17.93 (4.92)	<<1e-16
*APOE * ε 4 positive, no. (%)	383	87 (29.19%)	17 (33.33%)	13 (38.24%)	4.99e-01
*APOE* ε 2/ ε 2, no. (%)	306	2 (0.67%)	0 (0%)	0 (0%)	
*APOE* ε 2/ ε 3, no. (%)	306	27 (9.06%)	3 (5.88%)	0 (0%)	
*APOE* ε 2/ ε 4, no. (%)	306	4 (1.34%)	3 (5.88%)	0 (0%)	
*APOE* ε 3/ ε 3, no. (%)	306	129 (43.29%)	18 (35.29%)	10 (29.41%)	
*APOE* ε 3/ ε 4, no. (%)	306	75 (25.17%)	12 (23.53%)	11 (32.35%)	
*APOE* ε 4/ ε 4, no. (%)	306	8 (2.68%)	2 (3.92%)	2 (5.88%)	

^†^ *P*-values were calculated based on Mann–Whitney U-tests and using χ^2^ tests of independence for all binary variables.

The healthy individuals in the analysed cohort were, on average, 73.29 years of age (Standard deviation, SD = 7.53), while the MCI and AD dementia patients were 76.33 years (SD = 7.45) and 76.65 years (SD = 6.69) of age, respectively. Furthermore, women were over-represented in the healthy control group (67.11% female samples), but not in the MCI (49% female samples) and dementia (41% female samples) groups. Other available metadata in the cohort were the body mass index (BMI), MoCA scores, years of education, and *APOE* genotype. A lower BMI was observed in AD dementia patients compared to both the cognitively healthy and MCI participants. Furthermore, no difference in the years of education was observed between healthy, MCI, and AD dementia participants. The MoCA test scores showed a strong, consistent decline in cognitive abilities with dementia progression. Finally, a higher proportion of *APOE* ε 4 positive individuals was observed in the AD dementia patients (38.24%) compared to cognitively healthy individuals (29.19%). This increased proportion was not significant after testing for over-representation of *APOE* ε 4 carrier status (Methods, Table 1).

### 2.2. Generation of gut microbiome-personalized WBMs

To create the microbiome-personalized WBMs, we first mapped the species-level taxonomies in the gut metagenomics data onto microbial genome-scale metabolic reconstructions in the AGORA2^12^ and APOLLO resources,^13^ which contained 7,302 human microbial strains and 247,092 genome-specific metabolic reconstructions of human microbes, respectively. After taxonomic mapping, we renormalized the relative abundances and removed three additional microbial species that were below the relative abundance cut-off of one in a million in at least one sample. This procedure mapped a total of 430 out of 1,299 microbial taxa onto AGORA2 and APOLLO, which corresponded to an average read coverage of 71% (SD = 16%) compared to the pre-mapped metagenomic reads (Tables 2, S1). Of the 869 unmapped microbial taxa, 544 (62%) were not, or only partially, characterized at the species level. If the remaining 325 unmapped microbial species were mapped, the average read coverage would increase from 71% to 80% (Table S1).

**Table 2.** The effects of taxonomic mapping on the microbiome read counts and diversity metrics. The before-and-after mapping columns display the mean and standard deviation of read counts and diversity statistics before and after taxonomic mapping on the AGORA2 and APOLLO resources. The fold changes indicate the mean relative change after taxonomic mapping for the statistics. Fold-change values below one indicate a reduction in read counts and ecological diversity after taxonomic mapping. The Shannon and Simpson indices are diversity metrics that take into account both species richness and ecological evenness in a sample. The Shannon index is more influenced by species richness, while the Simpson diversity index is more influenced by ecological evenness. The lower diversity indices after mapping indicate that the taxonomic diversity is reduced as a result of the mapping procedure. The mean Shannon fold change is lower than the mean Simpson fold change, indicating that species richness might be more affected than ecological evenness.

Statistic	Before mapping	After mapping	Fold change	*P*-value^†^
Mean read counts (SD)	6,993,476 (2,706,890)	5,043,143 (2,431,164)	0.714 (0.16)	<<1e-16
Total number of named microbial species	1,299	430	0.33	
Mean species richness (SD)	149.48 (45.59)	61.272 (19.733)	0.46 (0.235)	<<1e-16
Mean Shannon index (SD)	3.101 (0.5)	2.529 (0.463)	0.843 (0.235)	<<1e-16
Mean Simpson diversity index (SD)	0.892 (0.08)	0.841 (0.106)	0.953 (0.169)	<<1e-16

^†^ *P*-values were calculated using a Mann-Whitney U-test.

Taxonomic mapping further reduced species richness from an average of 149.48 (standard deviation, SD = 45.59) to 61.27 (SD = 19.73; Table 2). The Shannon and Simpson diversity indices also showed a loss in microbial diversity after mapping. Notably, the relative decrease in the Simpson diversity index was less severe than the relative reduction in the Shannon index. This observation suggests that the ecological evenness could be less affected than the loss in taxonomic richness, as the Simpson diversity index is more influenced by ecological evenness than the Shannon index.^26^ Furthermore, taxonomic mapping changed the mean average relative abundances of the most abundant phyla, *Bacteroides* and *Firmicutes*, from 50.05% (SD = 19.44%) to 64.78% (SD = 20.36%) and from 35.86% (SD = 17.33%) to 21.46% (SD = 15.51%), respectively (Table S1). The mapping procedure also increased the sample relative abundances of the most abundant microbial species, *B. uniformis*, which changed from an average of 10.23% (SD = 12.15%) before mapping to 13.48% (SD = 14.99%) after mapping. After taxonomic mapping, the filtered metagenomic reads were used to create personalized microbiome community models, which were joined with the large intestinal lumen of sex-matched male and female WBMs (Methods). The generated microbiome-personalized WBMs (Table S2) were also given a diet corresponding to the metabolic intake of an average Western dietary intake^27^ (Table S3), which is the standard diet used for studies on host-microbiome co-metabolism that utilize microbiome-personalized WBMs.^22^ ^,^ ^23^ ^,^ ^28^ In summary, the microbiome-personalized WBMs captured on average 71.4% (SD = 16%) of the reads in the metagenomic data.

### 2.3. Selection of metabolites for *in silico* modeling

To predict microbial influences on metabolites linked with AD, we first selected a set of 38 metabolites for modeling. These metabolites were chosen as they were all previously investigated in WBM studies on microbial links to AD risk factors^22^ and AD status.^23^ The selected metabolites (Methods, Table S4) included twelve biochemical subclasses, of which “bile acids, alcohols, and derivatives” (18 metabolites) was the most abundant. Other abundant biochemical subclasses were “amino acids and peptides” (six metabolites) and benzenediols (four metabolites). All 38 selected metabolites were present in the WBM blood compartments, of which 22 could be produced by gut microbes in the WBMs. The remaining sixteen metabolites had metabolic precursors with microbial origins. Furthermore, eleven of the analysed metabolites were also present in the defined Western diet, while twelve metabolites could only be produced by gut microbial species (Table S4). Although the selected set of metabolites likely captured only a small part of all microbially influenced metabolites in AD patients, the 38 modeled metabolites were chosen to achieve our specific aims of validating previously predicted gut microbiome-driven metabolic associations with AD or risk factors of AD and for assessing the validity of negative findings in these previous modeling results.

### 2.4. Prediction of gut microbiome influences on AD-related metabolites in blood

Previously, we have shown that gut microbiome influences on L-arginine, S-adenosyl-L-methionine, deoxycholate, and lithocholate were associated with increased risk for AD.^22^ To test whether these prior findings may persist in MCI and AD dementia, we predicted the maximal metabolic flux for the accumulation of the selected metabolites in the blood of the microbiome-personalized WBMs. After processing the predicted fluxes, we performed a multivariate outlier inspection (Methods) and identified two outlier samples, which were subsequently removed. We then associated the predicted fluxes with cognitive status by performing logistic regressions on the flux predictions. Control variables were added for sex, age at stool collection, and relevant technical covariates (see Methods). The regressions were performed in a pairwise manner, i.e., healthy versus MCI, MCI versus dementia, and healthy versus dementia. Five metabolites were identified to potentially associate with MCI or AD dementia status. The flux predictions on S-adenosyl-L-methionine, L-arginine, and creatine were associated with both MCI and AD dementia. Furthermore, the taurine flux predictions were associated with MCI, but just missed significance for AD dementia (*p* = 0.09). Lastly, the formate flux predictions were associated with AD dementia status, but not with MCI status (Figure 2, Table S5). All five metabolites had higher predicted flux values in MCI or AD dementia compared to healthy controls. No changes between MCI and AD dementia patients were found in the flux predictions. However, this lack of change between MCI and AD dementia patients might be explained by all the analysed MCI patients also having a presumptive AD diagnosis. Nonetheless, the median values and regression estimates of the predicted fluxes were consistently higher in AD dementia patients compared to MCI patients, potentially indicating a consistent change with AD status. No significant associations with MCI or AD dementia status were detected for secondary bile acids. However, we did observe weakly increased predicted fluxes from healthy controls to MCI for deoxycholate (*p* = 0.08) and lithocholate (*p* = 0.09, Table S5). No such association was present for deoxycholate and lithocholate when comparing healthy controls and AD dementia patients. Taken together, we predicted increased potential gut microbiome contributions to S-adenosyl-L-methionine, L-arginine, creatine, taurine, and formate in blood. These results align with our prior findings^22^ and identify creatine and formate as circulatory metabolites with altered gut microbial influences in AD. Importantly, as these findings result from a targeted analysis, they are unlikely to represent the full spectrum of AD-associated gut microbiome influences on blood metabolites.

**Figure 2.**
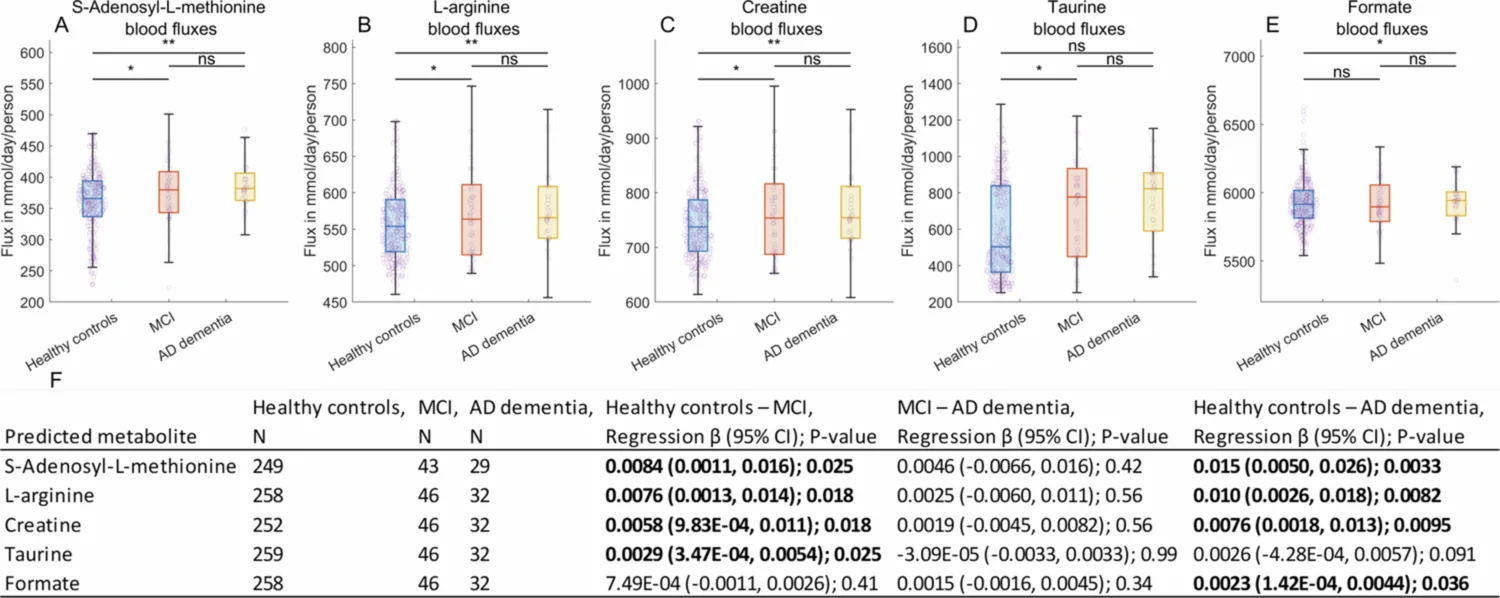
Predicted host-microbiome production capacities for circulatory metabolites. (A–E) Show the predicted fluxes in the blood of healthy controls (blue), MCI (red), and AD dementia patients (yellow) for the analysed metabolites that were associated with either MCI or AD dementia status. The asterisks above each box plot indicate the significance level. Two asterisks indicate that *P* < 0.01, one asterisk means that *P* < 0.05, while ”ns” indicates that no association was identified. (F) Shows the regression table summarizing the logistic regression results for the shown metabolites in A–E. The number of samples (*N*) for which fluxes could be predicted is shown for healthy controls, MCI, and AD dementia patients. Note that there were small differences in sample sizes between the analysed reactions, which were due to differences in the number of flux predictions with an optimal solution. For each regression fit, the regression β , 95% CI, and regression *p*-value are shown. The bold text indicates that the obtained *P-*value was below 0.05. The regression β shows the log odds ratios for the change in probability of a more severe AD state when increasing the blood flux values. The positive β values indicate that higher predicted blood fluxes are associated with more severe cognitive decline.

### 2.5. The predicted blood fluxes did not associate with plasma abundances from the same samples

We assessed whether the predicted metabolic fluxes would correlate with plasma metabolomic abundances from a subset of 168 individuals in the analysed cohort, including 130 healthy controls, 28 MCI patients, and 10 AD dementia patients. L-arginine, creatine, and taurine have been measured in the plasma samples, but not S-adenosyl-L-methionine and formate. To evaluate whether the flux predictions were predictive of the plasma abundances, linear regressions were performed (Methods) on the predicted fluxes against their corresponding plasma abundances. In line with previous observations,^22^ ^,^ ^23^ no association was found for the three tested metabolites (Table S6). We then assessed whether the relative plasma metabolomic abundances of L-arginine, creatine, or taurine associated with AD progression, as previously reported.^29-34^ However, no association with MCI or AD status was found for these metabolites in these samples (Table S6). This lack of signal in the metabolomic data may be explained by the lower sample numbers for the plasma metabolomics, as only 28 MCI and 10 AD dementia patients were included. Taken together, this analysis did not observe correlations between the *in silico* flux predictions and corresponding plasma metabolomics, concurring with previous observations.^22^ ^,^ ^23^

### 2.6. Identification of microbial contributors to blood fluxes associated with AD status

Next, we identified the microbial species that influenced the altered flux predictions in MCI and AD dementia patients. These flux-associated microbial species were identified by (1) utilizing the mechanistic structure of the WBMs to find the set of microbial species that influenced the predicted metabolites, and (2) by using regularized regressions to identify the sparsest set of microbial species that were most predictive of the fluxes (Methods). To identify which microbial species could have contributed to the predicted flux values, we analyzed the FBA shadow price solutions for the microbial species biomass compounds in each microbiome-personalized WBM. These shadow prices measured the sensitivity of the predicted fluxes to a hypothetical change in the abundance of a microbial species. For each predicted metabolite, the mean microbial species biomass shadow price values across all analysed samples were calculated, along with the associated 95% confidence intervals (CI) around the mean (Methods). Microbial species were then removed from the list of potential microbial influencers if their mean shadow price value was not statistically different from zero, i.e., the 95% CI around the mean microbial species biomass shadow price included zero. Out of the total 430 microbial species in the microbiomes, 303 potential microbial influencers of S-adenosyl-L-methionine were identified. For L-arginine and creatine, 286 and 289 microbial influencers were found, respectively, while for taurine and formate, 325 and 304 potential microbial influencers were found (Tables 3, S7).

**Table 3.** Summary statistics on the number of microbial species that limited the blood flux predictions for the selected metabolites. Columns two to four show the total number of potential microbial contributors, the mean, and the standard deviation of microbial contributors across the cohort. Columns five to seven show the same summary statistics for a filtered set of analysed microbial contributors to the predicted fluxes. The last column shows the explained variance of the analysed microbial species for the associated flux predictions. The explained variance was calculated after fitting elastic net regression models (see Methods) with the flux predictions as the response variable and predictors for the analysed microbial species and with sex as the control variable.

Metabolite	Identified microbial contributors			Analysed microbial contributors			
	Total	Mean	SD	Total	Mean	SD	Explained variance ( $R^{2}$ )
S-adenosyl-L-methionine	303	58.12	19.49	8	4.37	1.81	0.799
L-arginine	286	51.28	16.29	15	6.32	2.84	0.996
Creatine	289	54.84	17.19	16	6.83	3.11	0.996
Taurine	325	61.62	20.19	11	5.63	2.67	0.999
Formate	304	56.42	17.89	20	9.37	4.00	0.803

However, these microbial species could not have equally contributed to the flux predictions due to differences in their sample presence and relative abundances. To identify the most important microbial influencers of the predicted fluxes, we performed feature selection using the stability selection method,^35^ which applies LASSO regressions on bootstrapped samples^36^ to select the minimal set of microbial species that were most predictive of the flux predictions (Methods). This step drastically narrowed the list of flux-associated microbial species (Tables 3, S7). For S-adenosyl-L-methionine, eight key microbial species were identified, which, after controlling for sex, explained 79.9% of the variance in the S-adenosyl-L-methionine flux predictions (Methods). L-arginine and creatine had 15 and 16 flux-associated microbes, respectively, that could explain 99.6% of the variance in both L-arginine and creatine flux predictions. For taurine and formate, eleven and twenty key microbial species were identified that explained 99.9% and 80.3% of their associated fluxes after controlling for sex, respectively. To summarize, we utilized the microbe-associated shadow prices and a multivariate feature selection method to find potential key microbial species for each of the five metabolites with AD-associated fluxes. The identified sets of flux-associated microbial species were highly predictive of the flux results, confirming that the identified microbial sets contained major drivers of the flux predictions.

### 2.7. *B. uniformis* and *B. thetaiotaomicron* were key drivers of the increased metabolic flux predictions in AD patients

After identifying the set of microbial influencers of the flux predictions, we aimed to identify the key microbes driving the AD-associated flux predictions. To find these microbial species, we performed elastic net regressions^37^ (Methods), a multivariate method for identifying relevant features in omics datasets.^38^ The elastic net regressions were performed on the z-scaled flux predictions with covariates for the selected flux-associated microbes and sex as the control variable. These regressions identified *B. uniformis* as the most important microbial contributor to the AD-associated predicted metabolites, except for formate, which was highly influenced by *Escherichia coli* (Figure 3, Table S8).

**Figure 3.**
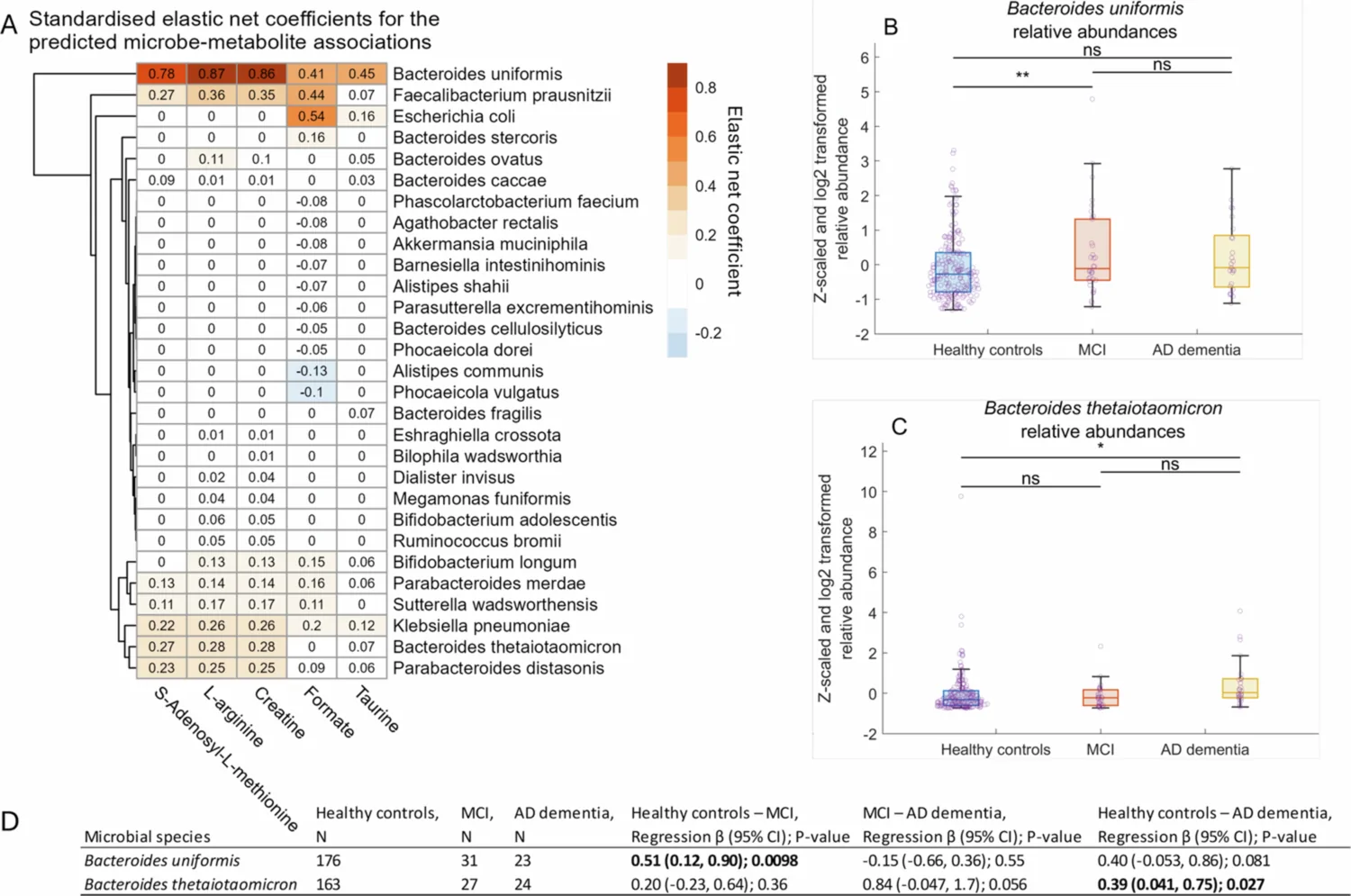
(A) Standardized elastic net coefficients for the selected microbial contributors to the predicted metabolites. The standardized coefficients represent the predicted change in flux value in standard deviations resulting from a change of one standard deviation in the microbial relative abundance. High positive coefficients (red) indicate a strong positive association between blood metabolic flux predictions and microbial species relative abundances. Conversely, negative coefficients (blue) suggest negative flux-microbe associations. Coefficients of zero indicate that a microbial species was not identified to be associated with the corresponding flux prediction. The elastic net coefficients show that *B. uniformis* is a key microbial contributor to the flux predictions of all analysed metabolites. (B and C) Box plots showing the microbial relative abundances of *B. uniformis* and *B. thetaiomicron* in healthy controls, MCI, and AD dementia patients, which were the two microbial contributors to the AD-associated flux predictions with altered relative abundances in MCI or AD dementia. Both microbial species had higher relative abundances in AD patients compared to controls. No other flux-associated microbial species was associated with MCI or AD status. The asterisks above each box plot indicate the significance level. Two asterisks indicate that *P* < 0.01, one asterisk means that *P* < 0.05, while ”ns” indicates that no association was identified. (D) Regression table summarizing the logistic regression results of the shown microbial species in B and C. The number of samples (N) in which each microbial species was present is shown for controls, MCI patients, and dementia patients. For each regression, the regression β , 95% CI, and regression *p*-value are shown. The bold text indicates that the obtained *P-*value was below 0.05. The regression β shows the log-odds ratios for the change in probability of a more severe AD state for higher relative abundances. The positive β values show that higher relative abundances of *B. uniformis* and *B. thetaiomicron* were associated with AD dementia.

*E. coli* was also associated with the predicted taurine blood fluxes, but did not associate with S-adenosyl-L-methionine, L-arginine, and creatine. Another major contributor to the predicted blood fluxes was *Faecalibacterium prausnitzii*, which was the second most important influencer of the flux predictions for all metabolites, except for taurine. Furthermore, we found *B. thetaiotaomicron* to be a key contributor to the predicted creatine, L-arginine, and S-adenosyl-L-methionine fluxes. Finally, the elastic net regression models found a negative coefficient for *Alistipes communis* with the formate flux predictions, indicating that increased growth of *A. communis* was associated with lower formate flux predictions. Next, we tested whether these microbial keystones were also associated with the AD stages by performing the same logistic analysis as described for the flux predictions (Methods). These logistic regressions found increased relative abundances of *B. uniformis* and *B. thetaiotaomicron* in AD dementia patients (Figure 3B–D, Table S8), suggesting that these microbes could have driven the increased flux predictions in MCI patients and AD dementia patients. Taken together, we identified the key microbial species influencing the AD-associated flux predictions and found increased relative abundances of the key microbes, *B. uniformis* and *B. thetaiotaomicron*, in MCI patients and AD dementia patients.

### The predicted AD-associated blood fluxes were also associated with *APOE* ε 2 and *APOE* ε 4 carrier status in healthy individuals

2.8.

Next, we tested whether the five identified AD-associated metabolites would also have different predicted fluxes in healthy individuals with lower or higher risk for developing AD. Therefore, we used only the 298 gut metagenomic samples from cognitively healthy individuals and investigated differences in the flux predictions in allelic risk groups for the *APOE* gene. There are three allelic variations of the *APOE* gene: ε 2, which protects against AD pathogenesis; ε 3, which is the normal risk allele; and ε 4, which is linked with increased risk for AD.^39^ ^,^ ^40^ These three alleles give rise to six allelic combinations ( ε 2/ ε 2, ε 3/ ε 3, ε 4/ ε 4, ε 2/ ε 3, ε 2/ ε 4, and ε 3/ ε 4). As the ε 2/ ε 2 and ε 4/4 allelic combinations had low abundances in our samples (Table 1), we combined the individuals with the ε 2/ ε 2 and ε 2/ ε 3 genotypes into the *APOE* ε 2 group. Individuals with ε 3/4 and ε 4/4 were conversely grouped into the high-risk ε 4 group. The normal-risk ε 3 group included only the *APOE* ε 3/ ε 3 genotype. Individuals with the *APOE* ε 2/ ε 4 genotype were excluded from this analysis due to the simultaneous expression and potential counteracting effects on microbiota of both the neuroprotective *APOE* ε 2 and the AD-risk-associated *APOE* ε 4 alleles. Multiple linear regressions were performed in a pairwise manner between two *APOE* groups (predictor), i.e., ε 2 versus ε 3, ε 3 versus ε 4, and ε 2 versus ε 4, and the blood flux predictions for the 38 analysed metabolites (Methods). The regressions found associations with the *APOE* risk groups for all analysed metabolites except for creatine, which narrowly missed the significance threshold (Figure 4, Table S9).

**Figure 4.**
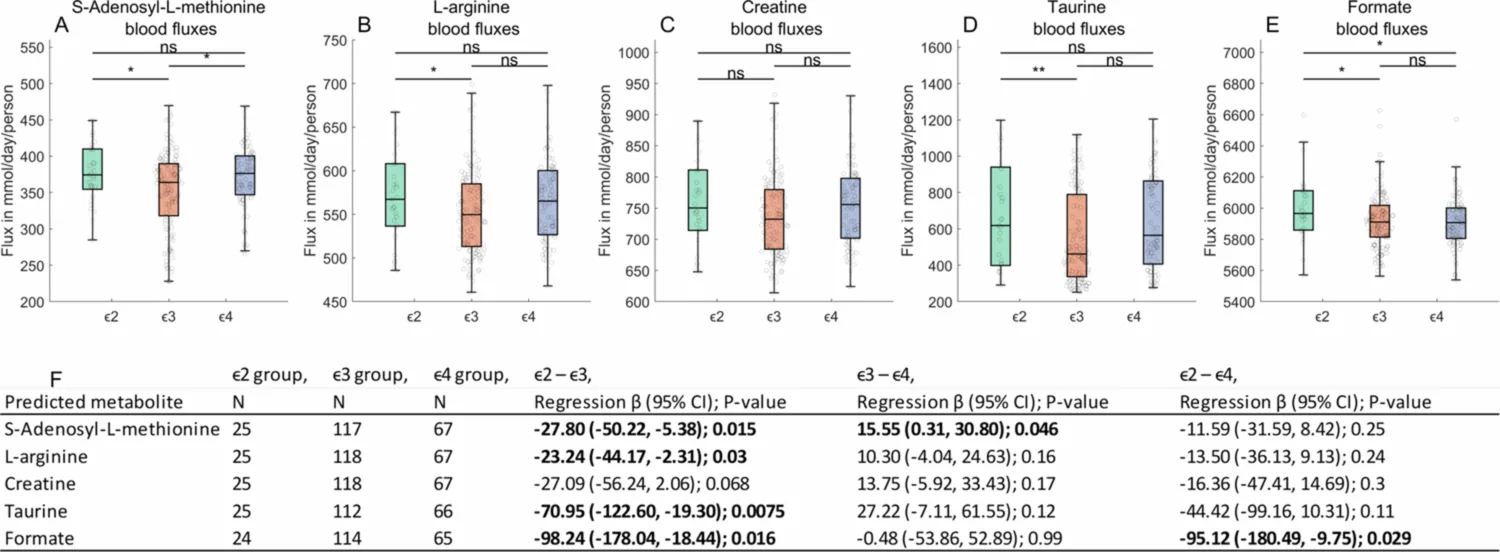
Predicted host-microbiome blood production capacities in healthy individuals for the selected circulatory metabolites. (A–E) shows the predicted blood fluxes for the ε 2 (green, ε 2/2 & ε 2/3), ε 3 (orange, ε 3/3), and ε 4 (blue, ε 3/4 & ε 4/4) *APOE* risk groups. The purple dots indicate the predicted flux values in each group. The asterisks above each box plot indicate the significance level. Two asterisks indicate that *P* < 0.01, one asterisk means that *P* < 0.05, while ns indicates that no association was identified. (F) Regression tables for the metabolites shown in A–E. Results are shown for each pairwise group comparison, i.e, ε 2 versus ε 3, ε 3 versus ε 4, and ε 2 versus ε 4. The results were obtained through pairwise multiple linear regressions on the *APOE* risk groups, while controlling for household and relevant technical covariates (Methods). The β and 95% confidence intervals (CI) show the average change in flux value from the first group, e.g., ε 2, to the second group, e.g., ε 3. The bold text indicates that the obtained *P*-value was below 0.05. Note that there were small differences in sample sizes between the analysed reactions, which were due to differences in the number of flux predictions with an optimal solution. These results show lower blood flux predictions in ε 3 for taurine, L-arginine, and S-adenosyl-L-methionine compared to both ε 2 and ε 4, and lower blood fluxes in the ε 4 group for formate compared to the ε 2 group.

When comparing the low-risk ε 2 group to the high-risk ε 4 group, differential fluxes were only found for formate, which had lower fluxes in the ε 4 group. When comparing the low-risk ε 2 group to the normal-risk ε 3 group, higher fluxes were found in the ε 2 group for S-adenosyl-L-methionine, L-arginine, and taurine, potentially indicating similar bacterial influencers of these metabolites. Finally, higher S-adenosyl-L-methionine flux predictions were found in the ε 4 group compared to the ε 3 group. We then assessed whether the previously identified key microbial drivers of the AD-associated flux predictions may have also driven the differential fluxes between the *APOE* groups. Therefore, we performed additional regressions to associate their relative abundances with *APOE*, as described for the flux predictions. These regressions could, however, not find any differences in relative abundances between the *APOE* risk groups for *B. uniformis* and *B. thetaiotaomicron*, suggesting that the differential fluxes across the *APOE* groups might not have been driven by these microbial species. We further investigated whether the analysed metabolites had altered fluxes in healthy individuals with differing MoCA scores. MoCA is a popular psychometric test with high sensitivity for detecting subjective and mild cognitive impairments.^41^ To analyze potential associations, we performed linear regressions on the predicted blood fluxes from healthy individuals (predictor) with the MoCA test scores as the response variable, while controlling for relevant confounding variables (Methods). However, none of the analysed metabolites correlated with MoCA test scores in the plasma metabolomics (Table S9). This lack of associations with the MoCA scores may, however, be explained by the analysed subcohort consisting of cognitively healthy individuals. To summarize, the analysed AD-associated flux predictions differed between the *APOE* risk groups in healthy individuals from the analysed cohort, but not with the MoCA test scores.

## 3. Discussion

In this study, we examined the metabolic influences of gut microbiota on circulatory metabolites that may influence the AD phenotype. To that end, we predicted the host-microbiome production capacities for blood metabolites in gut microbiome-personalized WBMs in healthy controls, MCI, and AD dementia. Our main results included (i) an increase in the predicted host-microbiome production capacities of S-adenosyl-L-methionine and L-arginine in MCI patients and AD dementia patients compared to healthy controls. (ii) We introduced a novel approach for identifying the key microbial contributors to predicted circulatory metabolites and determined that *B. uniformis* was the key contributor to the AD-associated blood flux predictions. (iii) We showed that the predicted AD-associated host-microbiome metabolite production capacities were also associated with *APOE* genotype in healthy individuals.

Our analysis revealed that the predicted gut microbiome contributions to S-adenosyl-L-methionine, L-arginine, and creatine in blood increased in MCI and AD dementia patients compared to healthy controls. Furthermore, we showed that formate flux predictions were increased only in AD dementia compared to healthy controls, while taurine flux predictions were only significantly increased in the MCI group. An increase in the taurine flux predictions was also identified when comparing healthy controls to the AD dementia group. However, this result did not reach significance. The flux predictions further showed no changes between MCI and AD dementia patients, suggesting that the relative abundances of the gut microbiota that influenced the analysed metabolites did not substantially differ between the MCI and AD dementia groups. This interpretation is supported by the finding that none of the major microbial contributors to the MCI- or AD dementia-associated metabolic flux predictions had differences in their relative abundances between the MCI and AD dementia groups (Figure 3). This result also aligns with previous systematic reviews by Ticinesi et al. (2018),^42^ and by Jemimah et al. (2022),^43^ who suggested that changes in gut dysbiosis may not always be identified between MCI and AD dementia.

We hypothesize that the predicted increase in microbial contributions to S-adenosyl-L-methionine could indicate a gut microbial influence on DNA methylation, as S-adenosyl-L-methionine is the main cofactor for DNA methylation.^44^ This interpretation would align with previous results from post-mortem brain samples, which identified increased S-adenosyl-L-methionine abundances in AD patients.^45^ ^,^ ^46^ However, lower cerebrospinal fluid levels of S-adenosyl-L-methionine have also been associated with a higher risk for AD progression in MCI patients.^47^ Lower S-adenosyl-L-methionine abundances have been previously proposed to result from vitamin B12 deficiency,^48^ which is associated with AD incidence.^49^ S-adenosyl-L-methionine is mainly sourced in the liver from L-methionine and ATP.^50^ Our result suggests that the gut microbiome may influence methionine metabolism by increasing the availability of its precursors. However, it remains difficult to ascertain how these microbial influences could alter plasma levels of S-adenosyl-L-methionine, as our models do not account for the complex regulatory feedback loops of methionine metabolism.^51^ The predicted increase in gut microbiome contribution to L-arginine in AD patients may suggest diverse gut microbial effects on human metabolism, as L-arginine is a key precursor for several metabolites that associate with AD pathology, such as nitric oxide, L-glutamate, GABA, polyamines, and creatine.^9^ L-arginine has been proposed to potentially mitigate cognitive decline by reducing oxidative stress.^52^ However, the effects of microbial influences on L-arginine metabolism may be diverse, as increased L-arginine abundances have also been associated with AD in post-mortem brain tissues,^53^ ^,^ ^54^ while lower L-arginine abundances in plasma have been linked with higher cognitive function in old age.^55^ Moreover, multiple gut microbiome-driven contributions to host metabolism may interact to produce deleterious effects. For example, we hypothesize that the higher gut microbiome contribution potential of S-adenosyl-L-methionine and L-arginine may influence the production of asymmetric dimethylarginine, which is formed from L-arginine and two methyl groups donated by S-adenosyl-L-methionine. Dimethylarginine is a known antagonist of nitric oxide production and has been previously linked to Alzheimer’s disease^56^ and cardiovascular dysfunction,^57^ ^,^ ^58^ which itself is a major risk factor for Alzheimer’s disease.^59^ The increased S-adenosyl-L-methionine and L-arginine fluxes may link with the predicted increased microbial influences of taurine and L-arginine. S-adenosyl-L-methionine is a precursor of taurine via the production of S-adenosyl-homocysteine, which is reduced to cysteine, which is in turn oxidized and decarboxylised to produce taurine.^60^ ^,^ ^61^ Conversely, creatine biosynthesis occurs through the production of guanidinoacetate from L-arginine and glycine, which is then methylated by S-adenosyl-L-methionine to produce creatine.^62^ Both taurine and creatine have important functions in human health. Creatine is the major source for phosphocreatine, which is the key cellular storage molecule for ATP.^63^ Taurine has functions in human physiology that range from bile acid detoxification^64^ to osmoregulation^65^ and blood pressure regulation.^66^ However, taurine has also been reported to protect against neurodegeneration by refolding misfolded proteins^67^ and can potentially mitigate amyloid β accumulation.^68^ ^,^ ^69^ In addition, previous plasma metabolomic studies identified a lower risk of AD with higher taurine blood abundances,^29^ ^,^ ^31^ making it unclear how the predicted increase in taurine production capacity by MCI gut microbiota may influence cognitive decline. Finally, our results included increased predicted microbial influences on formate. This result may suggest a microbial link with its direct precursor, formaldehyde, which is a neurotoxic compound and known antagonist of methylation.^70^ However, lower formate urine and fecal levels have also been reported in AD patients,^23^ ^,^ ^71^ suggesting a more complex and nuanced role of formate production in AD patients.

In our prior study, we associated predicted blood fluxes of L-arginine, S-adenosyl-L-methionine, deoxycholate, and lithocholate with increased risk for AD in healthy individuals.^22^ We validated these prior predictions by showing that L-arginine and S-adenosyl-L-methionine were also associated with MCI and AD. Although no significant signal was identified for deoxycholate and lithocholate blood fluxes, we did observe a tendential increase in the flux predictions for these metabolites in AD patients (Table S5), which is in line with the mounting evidence for increased microbial conversion of bile acids in AD progression from MCI.^5^ ^,^ ^72^ ^,^ ^73^ Taken together, our targeted analysis confirmed previous *in silico* predictions and expanded the set of circulatory metabolites, which may be differentially influenced by gut microbiota in AD.

In this study, we further assessed whether the predicted fluxes associated with cognitive status could be validated in the plasma metabolomics data. However, no associations with AD or *APOE* allele groups could be found in the assessed plasma metabolomics. Crucially, this validation analysis had several major limitations. First, S-adenosyl-L-methionine and formate, which had increased flux predictions in AD dementia patients, were absent from the plasma metabolomic dataset, and thus could not be investigated. In addition, only 168 of the 383 analysed gut microbiome samples had corresponding metabolomic data, which included only ten AD dementia patients, thus making the validation analysis severely underpowered. Validation efforts may be improved in the future by ensuring matched sampling of plasma and stool samples and by selecting *in silico* metabolic objectives based on the detected metabolites in the metabolomic dataset. Furthermore, we also could not identify significant associations between predicted fluxes and plasma metabolomic data, which is in line with previous observations from whole-body modeling studies.^22^ ^,^ ^23^ However, it should be noted that the predicted blood fluxes and plasma abundances represent very different metrics of metabolism. Therefore, the missing correlations may be expected. The predicted fluxes were mainly driven by the gut microbiome compositions and represented the theoretical maximum capacity of the gut microbiomes to contribute to metabolites in the blood, while the plasma metabolomics represented the *in vivo* steady-state circulating metabolite abundance. Previous reports have also shown that direct correlations between gut metagenomic data and plasma metabolomics are challenging to establish.^24^ ^,^ ^25^ ^,^ ^74^ This difficulty in linking plasma metabolomics and gut microbiome data may be explained by several factors. For instance, plasma abundances and gut microbiome relative abundances are influenced by several independent factors. The gut microbiome composition is highly dependent on the gut environment and gut barrier integrity, which are, at least not directly, related to plasma metabolomic abundances. In contrast, the plasma metabolome is influenced by tissue uptake rates, systemic clearance, and simultaneous metabolic contributions from multiple organs. In addition, genetic and environmental interactions between the gut microbiota and plasma metabolomics may also differ, further preventing clear correlations. Moreover, the predicted maximal flux capacities may not necessarily correlate with changes in the actual metabolic activity of the gut microbiota. It has been previously shown that the gut metatranscriptome is more dynamic than the gut metagenome and that only a subset of possible pathways in gut microbes is active.^75^ Thus, the predicted flux solutions may have included active microbial pathways that were inactive in the actual gut microbes. Another possible explanation for the absence of flux–plasma associations is that the five identified metabolites are either common dietary compounds or predominantly synthesized *de novo* in humans. For example, creatine and taurine can be obtained from animal-source foods,^76^ meaning that plasma alterations may reflect dietary differences between healthy individuals and AD patients. Circulating S-adenosyl-L-methionine is furthermore primarily sourced from hepatic synthesis,^50^ whereas L-arginine levels may also be driven by increased protein catabolism or altered endogenous synthesis.^77^ Similarly, circulating formate concentrations could be explained by altered *de novo* synthesis from the folate cycle.^78^ Because the plasma abundances of these metabolites can be explained by non-microbial sources, shifts in gut microbiome contributions may not directly translate into measurable plasma changes. Nonetheless, microbial contributions could still exert effects on disease-associated molecular pathways and thereby influence AD progression. To circumvent the complexities of linking gut microbiome influences on human metabolism through *in vivo* measurements, future *in silico* modeling efforts on host-microbiome co-metabolism may benefit from experimental validation in gnotobiotic animal models^79^ or microfluidic gut-on-a-chip systems.^80^

We also identified the key microbial contributors to the five metabolites with altered blood fluxes in AD. To achieve this result, we developed a two-step feature selection strategy that utilized the mechanistic structure of microbiome-WBMs and statistical feature selection to reduce the search space of possible microbe-metabolite associations. This approach was highly effective in identifying the most predictive microbial contributors to the flux predictions, while remaining mechanistically grounded and accounting for the modeled dynamics of host–microbiome co-metabolism. Our analysis revealed that *B. uniformis* and *B. thetaiomicron* were major drivers of the increased flux predictions in MCI and AD dementia. *B. uniformis* is a commensal gut bacterium with reported probiotic properties.^81^ ^,^ ^82^ Similarly, higher relative abundances of *B. thetaiomicron* were identified as strong contributors to the elevated flux predictions in AD patients. This species is also a well-known commensal with potential probiotic effects, including reducing oxidative stress^83^ and alleviating ulcerative colitis symptoms.^84^ In addition, *E. coli* was identified as a major contributor to formate flux predictions (Figure 3). Although *E. coli* itself was not associated with AD status, it has been implicated in AD pathology due to its ability to produce amyloid- β peptides, such as curli fibers, which promote amyloid- β aggregation.^85^ Taken together, our feature selection strategy identified *B. uniformis* and *B. thetaiomicron* as key microbial contributors to the five analysed circulatory metabolites. While their roles in AD-related metabolic perturbations require further validation, these findings implicate both species as potential microbial targets for future therapeutic strategies.

Lastly, we observed that the metabolites with altered microbial contribution potentials in MCI and AD dementia were also associated with *APOE* genotype in healthy individuals. Whether the allelic variations of the *APOE* gene exert causal effects on the microbiome or whether the associations are purely correlational remains uncertain. Nevertheless, growing evidence supports a link between *APOE* genotype and gut microbial composition.^86-90^ Notably, our observed associations with *APOE* carrier status replicate our previous findings on S-adenosyl-L-methionine in healthy individuals,^22^ reinforcing a possible connection between *APOE* genotype and gut microbiome interactions with human methionine metabolism. More broadly, these findings are consistent with the hypothesis that gut microbial dysbiosis may arise before the onset of measurable cognitive decline,^91^ which would make the gut microbiome a potential early marker of dementia, as demonstrated previously.^92^

### 3.1. Strengths and limitations

The main strength of this study lies in the use of unique metabolic modeling methods, a richly annotated shotgun metagenomics dataset, and the availability of associated plasma metabolomics to investigate the metabolic effects of gut microbiota in AD patients. The combined mapping on both the AGORA2^12^ and APOLLO^13^ resources further ensured a high read coverage of the gut microbiomes in the metabolic models, enabling more accurate investigations of host-microbiome co-metabolism in AD patients. Nevertheless, this study contained several important limitations. For instance, matched plasma metabolomics were available for 168 of the 383 analysed gut microbiome samples, which has reduced the statistical power to identify plasma metabolomic associations with AD status. Another limitation was that, despite the improved mapping coverage compared to our prior study, ^22^ 28.6% of reads from named microbial taxa remained unmapped. This loss of taxa may have introduced biases in the flux predictions, such as a bias towards well-characterized microbial taxa in the AGORA2^12^ and APOLLO^13^ resources, which could have compounded upstream methodological limitations and biases relating to sample processing, gene sequencing, and incomplete taxonomic classifications. Furthermore, no dietary information was available for this study. As diet is a key variable influencing the gut environment, unknown dietary differences between AD patients and healthy controls may have confounded the results. Another issue resulting from the lack of dietary information was that the choice of the average Western diet may not have been representative of the average participant’s dietary intake of the cohort. Thus, our findings may not be robust when modeling diets that are dissimilar from the average Western diet, such as vegetarian or vegan diets. Future studies may generate improved modeling predictions by generating personalized metabolic dietary formulations from food-frequency questionnaire data.^93^ Another limitation was that no information was available on the metabolic activity of the gut microbiomes. This lack of measured microbial metabolic activity means that the *in silico* predicted metabolic capacities of the gut microbiota to influence host metabolism may not manifest *in vivo* due to factors of the gut environment, such as microbial nutrient availability and gut barrier integrity. The *in vivo* metabolic functions of gut microbiota may be more accurately modeled through the integration of metatranscriptomic data with the *in silico* models.^94^ Furthermore, the modeling predictions were limited by inherent features of the COBRA framework, such as the assumption of steady-state metabolism, which does not capture the dynamics of host-microbe and microbe–microbe interactions. In addition, the models could not capture the physiological changes of gut dysbiosis in AD patients, such as increased intestinal permeability.^95^ Previously, physiologically based pharmacokinetic models have successfully modeled gut microbiome-mediated intestinal metabolic absorption^96^ and have been integrated with WBMs.^97^ Future studies might integrate physiologically based pharmacokinetic models with microbiome-personalized WBMs to improve modeling predictions on gut microbial contributions to metabolites in the bloodstream.

### 3.2. Conclusions

In conclusion, we predicted shifts in potential gut microbiome contributions to circulatory metabolites that may influence AD pathology. We also determined that these altered metabolic predictions may be explained by increased relative abundances of *B. uniformis* and *B. thetaiomicron* using a novel feature selection and microbe-metabolite association approach. We further showed that AD-associated microbial contributions to the analysed predicted metabolites were also associated with *APOE* genotype in healthy individuals, suggesting a potential link between *APOE*, the gut microbiome, and AD pathogenesis. Although experimental validation will be necessary to confirm these predicted microbe-metabolite associations in AD, we hope that these results will help guide and inspire future efforts to uncover the key mechanisms of host-microbiome co-metabolism in AD dementia.

## 4. Materials and methods

### 4.1. Participant recruitment

Participants were recruited from ten NIA-funded Alzheimer’s Disease Research Centres that were part of the Alzheimer Gut Microbiome Project (AGMP), including UC San Diego, UC Davis, Stanford University, Kansas University, Wisconsin University, Indiana University, New York University, Wake Forest University, Cleveland Clinic, Nevada, and the University of Alabama at Birmingham. The stool collection kits were mailed to participants from the centers, and all kits were delivered to the National Centralized Repository for Alzheimer’s Disease and Related Dementia (NCRAD), where they were processed and sub-aliquoted before being banked or distributed. Participants were from all over the country and differed by race, geographical location, and socioeconomic status. Before admission into the ADRCs, participants provided written informed consent following the Declaration of Helsinki. After admission, participants underwent neuropsychological testing according to the standardized UDS 3.0 framework, which included the MoCA assessment. *APOE* genotypes were obtained from the whole-blood samples using competitive allele-specific polymerase chain reaction (PCR)-based KASP genotyping assays (LGC Genomics, Beverly, MA).^98^ Presumptive etiologic diagnoses of MCI due to AD and dementia due to AD were made by a multidisciplinary diagnostic panel and were based on the diagnostic criteria from the National Institute on Aging–Alzheimer’s Association.^99^ ^,^ ^100^

### 4.2. Metabolomics

Plasma samples were collected by the NCRAD fluid biomarker program on behalf of the AGMP. The plasma metabolomics data were generated from EDTA plasma samples by Metabolon Inc. (Durham, NC, USA). The raw plasma dataset included 1,665 biochemicals, 1,316 compounds of known identity (named biochemicals), and 349 compounds of unknown structural identity (unnamed biochemicals). After plasma sample collection, the samples were immediately stored at -80 degrees Celsius, after which they were prepared using the MicroLab STAR® system from the Hamilton Company. During the sample preparation, small molecules attached to proteins were recovered by first dissociating the small molecules bound to the proteins and then precipitating the proteins with methanol by shaking for 2 min (Glen Mills GenoGrinder 2000) and centrifugation. The extracts were then divided into five different aliquots. One aliquot was used for each of four analysis methods and one aliquot as a backup. Then, organic solvents were removed by placing the samples on a TurboVap® (Zymark), after which they were stored overnight under nitrogen.

The dried sample aliquots were all analyzed using Waters ACQUITY ultra-performance liquid chromatography (UPLC) and a Thermo Scientific Q-Exactive high-resolution/accurate mass spectrometer interfaced with a heated electrospray ionization (HESI-II) source and Orbitrap mass analyzer operated at 35,000 mass resolution. After overnight storage, the sample extracts were reconstituted in solvents compatible with each of their associated analysis methods. One sample aliquot was analysed in acidic positive ion conditions optimized for more hydrophilic compounds (PosEarly). The extract from this aliquot was gradient eluted from a C18 column (Waters UPLC BEH C18-2.1 × 100 mm, 1.7 µm) using water and methanol, containing 0.05% perfluoropentanoic acid (PFPA) and 0.1% formic acid (FA). Then, a second aliquot was analyzed using the same acidic positive ion conditions, but optimized for more hydrophobic compounds (PosLate). Gradient elution was performed from the same C18 column using methanol, acetonitrile, water, 0.05% PFPA, and 0.01% FA, but was operated at an overall higher organic content. A third aliquot was analyzed using basic negative ion optimized conditions and a separate dedicated C18 column (Neg). The basic extracts of this aliquot were gradient-eluted from the column using methanol and water, however, with 6.5 mM Ammonium Bicarbonate at pH 8. The fourth aliquot was analyzed using negative ionization following elution from a HILIC column (Waters UPLC BEH Amide 2.1 × 150 mm, 1.7 µm) in a gradient consisting of water and acetonitrile with 10 mM Ammonium Formate, pH 10.8 (HILIC). The MS analysis was performed by alternating between MS and data-dependent MSn scans using dynamic exclusion. The scan range covered 70-1000 m/z. The raw data were then extracted, peak-identified, and QC-processed using Metabolon’s hardware and software. Peak quantification was performed by calculating the area under the curve. A data normalization step was further performed to correct for variation from instrument inter-day tuning differences. In this step, each compound was corrected by registering the medians to equal one and normalizing each data point proportionately in run-day blocks.

After obtaining the corrected metabolomic peaks, metabolites with over 25% missing data points were excluded from the analysis. To account for sample variability, probabilistic quotient normalization was applied, followed by a log2 transformation. The remaining missing values were then imputed using the k-nearest neighbour algorithm. Outlier samples were identified and removed using the local outlier factor method implemented in the bigutilsr R package. To address extreme single concentration values, any measurement with an absolute abundance exceeding q = abs(qnorm(0.0125/n)), where *n* is the number of samples, was marked as missing. This threshold q corresponds to values with less than a 2.5% two-tailed probability of originating from the same normal distribution as the other measurements, incorporating a Bonferroni-like correction (by dividing by the sample size). The newly marked missing values were then imputed in a second round using the k-nearest neighbour algorithm. Batch effects were corrected using the COMBAT package.

### 4.3. Gut metagenomics

The analysed fecal samples were collected between March 2022 and June 2023 as previously described.^101^ Briefly, the samples were collected at home using standardized fecal collection kits. Collected samples were returned the same day via overnight delivery in insulated containers that were chilled with frozen gel packs. Upon return, the samples were weighed, scored on the Bristol stool scale, and stored at -80 degrees Celsius until further processing.

Sample DNA extraction was performed using the MoBio PowerMag Soil DNA isolation kit with a magnetic bead plate. The extracted genomic DNA (gDNA) was quantified using the Quant-iT PicoGreen double-stranded DNA (dsDNA) assay kit from Thermo Fisher Scientific Inc. The samples then underwent miniaturized KAPA HyperPlus library preparation using the iTru indexing strategy.^102^ ^,^ ^103^ The prepared libraries were normalized, pooled based on their concentrations, and PCR-cleaned. Size selection was performed based on the 300-700 bp range using the Sage Science PippinHT pipeline. DNA sequencing was then performed on an Illumina NovaSeq 6000 as a paired-end 150-cycle run at the University of California, San Diego (UCSD) IGM Genomics Centre.

The generated sequence data were then processed, trimmed, and filtered using the qp-fastp-minimap2 pipeline version 2023.12,^104^ ^,^ ^105^ as described previously.^106^ The reads were filtered using minimap2 against GRCh38.p13, T2T-CHM13v2.0, viral Phi X 174, and the Human Pangenome Reference assemblies.^107^ The processed metagenomic sequencing data were then uploaded to Qiita^108^ (Study ID: 15448) for further processing. Taxonomic classification was performed using the Woltka pipeline^109^ version v0.1.4 (qp-woltka 2023.11), which mapped the metagenomic data against the Web of Life 2 reference database. Lastly, the SCRuB^110^ pipeline was used to statistically correct the processed read table for well-specific microbial contamination and well-to-well sample leakage across the 96-well plate.

### 4.4. Taxonomic mapping

The processed gut metagenomics data were mapped onto pan-species genome-scale metabolic reconstructions in the combined AGORA2^12^ and APOLLO^13^ resources, using the MARS pipeline.^111^ After the mapping procedure, the metagenomic reads were normalized by calculating the relative read abundances per sample. To limit false positives in the mapped microbiomes, sample relative abundances below the threshold of 0.00001% were removed, and the remaining read abundances were re-normalized. The mapping coverages, i.e., the fraction of available metagenomic data in a sample after mapping, were calculated from the original and mapped relative abundances after removing reads with relative abundances below the threshold. After taxonomic mapping, an additional quality control step was performed, which resulted in samples being removed if they had fewer than 1 million reads after taxonomic mapping or a mapping read coverage below 25%.

### 4.5. Microbial metabolic reconstructions

Genome-scale metabolic reconstructions for the mapped microbial species were retrieved from the AGORA2 (version 2.01, https://www.vmh.life/files/reconstructions/AGORA2/) and APOLLO^13^ (version 1.0, https://doi.org/10.7910/DVN/PIZCBI) resources. As the metabolic reconstructions in AGORA2 and APOLLO are strain-resolved, pan-species models were generated using the *createPanModels.m* function.^112^ The created pan-species models represented the combined metabolic content of their associated strain-resolved reconstructions and contained the union set of all reactions, metabolites, and genes in the species-associated strain-level reconstructions. Furthermore, the pan-species models contain a new pan-species biomass reaction that was created by taking the union set of consumed metabolites in the associated strain-level reconstructions. For consumed metabolites present in more than one biomass reaction in the associated strain-level reconstructions, the mean stoichiometric coefficient was calculated for the consumed metabolite in the pan-species biomass reaction.

### 4.6. Microbiome community models

Personalized metabolic models of gut microbiome communities were created using the mgPipe module in the Microbiome Modelling toolbox 2.0.^113^ In mgPipe, personalized microbiome community models are created by joining the mapped pan-species models from a sample in a shared microbiota lumen and adding transport reactions to enable metabolic exchanges between the pan-species models and the shared microbiota lumen. Then, a dietary compartment and fecal compartment are connected to the microbiota lumen compartment to enable dietary intake and fecal excretion of microbial products. Next, the mapped relative abundances were integrated into the microbiome communities via the addition of a community biomass reaction (VMH reaction ID: communityBiomass), which lists each microbe’s biomass reaction with the microbe’s relative abundance as the stoichiometric coefficient. This community biomass reaction ensured proportional microbial growth for each integrated pan-species model per the mapped relative species abundances. Consequently, the community biomass reaction ensured that the metabolic contribution capacity of each microbial species to the host was proportional to their relative abundances.

### 4.7. Whole-body metabolic models

We used the generic male and female WBMs^20^ (version 1.04c^28^ for the host component of the microbiome-personalized WBMs. These organ-resolved WBMs captured the metabolism of 26 organs and 13 biofluid compartments, which were connected in an anatomically accurate manner. The male generic WBM contained 83,395 reactions, 58,095 metabolites, and 105,936 constraints, while the female generic WBM contained 85,892 reactions, 60,537 metabolites, and 109,757 constraints. All parameters were set to their defaults.^20^

### 4.8. Microbiome-personalized WBMs

The microbiome-personalized WBMs were created using the *combineHarveyMicrobiota.m* function, which integrates the personalized microbiome community models with the large intestinal lumen compartment of the generic WBMs, as described previously.^20^ This function also introduces transport reactions to enable metabolic exchanges between the microbiota and the host. This function also updated the coupling constraints for each microbe’s reaction to its biomass reactions, to a ratio of 1:400, following previous WBM modeling studies.^20^ ^,^ ^22^ ^,^ ^23^ ^,^ ^114^

Further parameterization of the WBMs was performed by setting the upper and lower flux bounds of the *Excretion_EX_microbiota_LI_biomass[fe]* reaction to one mmol/day/person. This reaction excretes the *microbiota_LI_biomass* compound, which is produced by the *communityBiomass* reaction. Fixing the flux bounds of the *Excretion_EX_microbiota_LI_biomass[fe]* reaction ensured that all generated WBMs produced the same metabolic flux over their microbiome community, regardless of differences in community compositions. In addition, the upper and lower flux bounds of the *Whole_body_objective_rxn* were set to one mmol/day/person, following previous WBM studies.^20^ ^,^ ^22^ ^,^ ^23^ ^,^ ^115^ The *Whole_body_objective_rxn* includes a linear combination of the whole-body organ biomass productions and associated coefficients for the proportional organ weights. This additional constraint ensured proportional organ biomass production between samples, corresponding to the organ weights.

### 4.9. Diet

The microbiome-personalized WBMs were further parameterized with dietary uptake reactions and reaction constraints from the Virtual Metabolic Human database (https://www.vmh.life).^27^ Reactions and constraints were chosen for the average Western diet, since sufficient information on dietary uptake was not available. This diet was selected because it was identical to the diet formulation used in prior studies that utilized microbiome-personalized WBMs,^22^ ^,^ ^23^ ^,^ ^28^ making the results of this study more comparable to those from previous studies. The dietary uptake formulation represented the metabolite-level average intake of a one-day meal plan for a 70 kg adult^27^ and was added to the models using the *setDietConstraints.m* CobraToolbox function. The applied diet contained 192 metabolites (Table S3), of which 92 metabolites were present in trace amounts below 0.1 mmol/day/person. The sensitivity of changes in the consumption of these 192 metabolites towards the flux predictions was further recorded and described by calculating the mean average reduced cost values of the dietary contents towards the flux predictions (Table S10). The reduced cost values of dietary reactions describe the linear effects on the blood flux predictions when minimally changing the lower flux bound of a dietary reaction, while holding everything else fixed.^116^ Note that, as reduced cost values represent the prediction sensitivity to single-parameter variations, they may not be informative of the effects on flux predictions due to dietary variations that involve multiple metabolic compounds.

### 4.10. Simulations

The metabolic fluxes in blood were predicted by performing FBA, which converts a genome-scale metabolic reconstruction into a mathematical model and interrogates the model using a linear programming solver according to an objective function.^14^ The objective function describes a linear optimization problem for finding extreme points in a constrained solution space of metabolic states as follows:

(1) $$\begin{array}{l} \begin{array}{cl} \underset{v}{max}\; & c^{T}\cdot v\\ s.t, & S\cdot v=0,\\ & lb\leq v\leq ub \end{array} \end{array}$$

The linear programming solver then finds a reaction flux vector, *v*, containing a maximized metabolic flux through an objective reaction, defined by a non-zero coefficient in *c*. The maximized flux value must lie within the feasible solution space defined by constraints, such as the steady state constraint. Steady state is defined by the system of linear equations, S⋅v=0 , which enforces unchanging metabolite concentrations over time. Consequently, the amount of produced metabolites (products) equals the amount of consumed metabolites in each reaction (substrates). The metabolic products and substrates in each reaction are encoded by non-zero coefficients in the stoichiometric matrix, *S*. Substrates are represented by a negative coefficient, while reaction products are indicated by positive coefficients. In addition to the steady-state constraint, each reaction in *v* was constrained by reaction-specific upper (*ub*) and lower (*lb*) bounds, derived from literature sources, such as thermodynamic data and experimental measurements. If no reaction information was available, reactions were left unconstrained by setting *lb* and *ub* to arbitrary values of −1,000,000 and 1,000,000 mmol/person/day (see also Thiele et al.^20^ for details). For the objective reactions, we introduced demand reactions into the WBM blood compartments (VMH name: [bc]) for each of the analysed blood metabolites. Demand reactions are unbalanced, left-sided reactions (e.g., DM_leu_L[bc]: 1 leu_L[bc] → Ø for L-leucine in blood), commonly used to predict the maximal potential metabolite productions in a system. In previous studies, demand reactions have been used in WBMs to predict blood biomarkers of single gene defects^117^ and gut microbiome flux influences associated with risk markers of Alzheimer’s disease.^22^ Demand reactions were separately introduced for each FBA using the *addDemandReaction.m* CobraToolbox function.^112^ FBA was then performed for each model by running *fba = optimizeWBModel(model)* with the *model.osenseStr = ’max’* parameter to ensure maximization of the flux through the objective reaction. Default parameters were used for the linear optimizations. After performing FBA, the maximized fluxes were extracted from the *fba.f* solution field and rounded to six decimals to remove potential numerical artifacts that can appear in small numbers.

### 4.11. Outlier removal

Outlier samples in the predicted fluxes were identified using a robust principal component analysis (PCA) on the z-transformed fluxes, performed with the alternating least squares algorithm. Potential outliers were identified by taking the Euclidean norm of the principal component scores for each sample, weighted by the explained variance of each principal component. This approach obtains the average distance of a sample in a principal component from the center of the flux data. Values close to zero indicate samples for which average fluxes were predicted, while the highest calculated values in the cohort were flagged as potential outlier samples across the predicted fluxes. In combination with a visual inspection of the first three PCA components, the ranked outlier scores were used to manually identify outlier samples to be removed.

### 4.12. Statistical analyses

#### 4.12.1. Study cohort descriptions

Fisher’s exact test was used to quantify sample differences in AD patients and controls for the binary variables in the metadata. The *fishertest.m* function was used to calculate the Fisher exact *p*-values, the associated odds ratios, and the 95% confidence intervals. For the numerical metadata, including age in years and the mapped read count, differences between AD patients and controls were tested by performing the Wilcoxon rank-sum test, using the *ranksum.m* MATLAB function.

#### 4.12.2. AD dementia analysis

Multiple logistic regressions were performed to identify differences in AD status (response) as a function of the predicted blood flux values (predictor) . The logistic regressions were stratified for pairwise investigations on each AD status group comparison, i.e., healthy control versus AD MCI, AD MCI versus AD dementia, and healthy control versus AD dementia. The logistic regressions further included sex and age at stool sample collection (Z-transformed) as household control variables. BMI was considered as a covariate, but was dropped due to 36 samples missing BMI information. Years of education and *APOE* ε 4 status were also considered as covariates, but were dropped, as no difference in education or *APOE* ε 4 was observed between the healthy controls, MCI, and AD dementia groups. Furthermore, technical control variables were added for the sequencing lane, whether ethanol was added during the DNA extraction (yes/no), and the total reads in each microbiome-personalized WBM (log10-transformed and Z-transformed). The same set of multiple logistic regressions was performed to associate the gut metagenomics reads in the microbiome-personalized WBMs with cognitive status and the plasma metabolomic abundances (log-transformed and Z-transformed). These regressions did not include control variables for the microbiome-specific covariates, including the sequencing lane, whether ethanol was added during the DNA extraction (yes/no), and the total reads in each microbiome-personalized WBM.

#### 4.12.3. Flux to plasma metabolomic association analysis

To assess the associations between predicted blood fluxes and the corresponding plasma metabolomics, linear regressions were performed on the log2-transformed and z-scaled flux predictions (predictor) against the z-scaled and log-transformed metabolomic plasma abundances (response) with control variables for sex, age, the total reads in each microbiome-personalized WBM (log10-transformed and Z-transformed), and the sequencing lane.

#### 4.12.4. *APOE* risk group analysis

Multiple linear regressions were performed to identify differences in the predicted blood flux values (response variable) across *APOE* risk group status (predictor). The linear regressions were stratified by pairwise investigations for each *APOE* risk group comparison, i.e., ε 2 versus ε 3, ε 3 versus ε 4, and ε 2 versus ε 4. Each linear regression included sex and age at stool sample collection (Z-transformed) as household control variables. Furthermore, technical control variables were added for the sequencing lane, whether ethanol was added during the DNA extraction (yes/no), and the total reads in each microbiome-personalized WBM (log10-transformed and Z-transformed). The same set of multiple linear regressions was performed to investigate associations between the gut metagenomics reads in the microbiome-personalized WBMs and the plasma metabolomic abundances (log-transformed and Z-transformed). However, the regressions on the plasma metabolomics did not include control variables for the microbiome-specific covariates, such as the sequencing lane, whether ethanol was added during the DNA extraction (yes/no), and the total reads in each microbiome-personalized WBM.

#### 4.12.5. MoCA score analysis

Multiple linear regressions were performed on the Z-scaled MoCA scores (response) with the predicted blood flux values as the predictor of interest. These linear regressions included age and sex as household covariates and technical covariates for the sequencing lane, whether ethanol was added during the DNA extraction (yes/no), and the total reads in each microbiome-personalized WBM (log10-transformed and Z-transformed). Additional control variables included BMI in kg/ $m^{2}$ (log10-transformed and Z-transformed), the number of years of education, and *APOE* ε 4 carrier status (yes/no) were added as control variables. The same set of multiple linear regressions was performed to investigate the gut metagenomics reads in the microbiome-personalized WBMs and the plasma metabolomic abundances (log-transformed and Z-transformed). However, the regressions on the plasma metabolomics did not include control variables for the microbiome-specific covariates, including the sequencing lane, whether ethanol was added during the DNA extraction (yes/no), and the total reads in each microbiome-personalized WBM.

### 4.13. Identification of key microbes for the flux predictions

#### 4.13.1. Shadow price analysis

Potential microbial influencers of the predicted fluxes were identified by analyzing the shadow prices in the FBA solutions associated with the flux predictions. Shadow prices are solutions to the dual linear problem and represent the sensitivity of the flux prediction to a change in metabolite availability. More precisely, the shadow price of a metabolite *i*, $\pi_{i}$ , represents the inverse ratio of an incremental relaxation of the steady state constraint for a metabolite *i*, $\delta b_{i}$ , and the resulting maximal change in the flux through the objective reaction, δZ :

(2) $$\pi_{i}=-\frac{\delta Z_{i}}{\delta b_{i}}$$

To identify the potential microbial influencers of the flux predictions, we analyzed the shadow prices of the microbial biomass metabolites in the WBMs. Microbial biomass metabolites were coupled with the metabolic reactions in the microbial pan model via flux capacity (coupling) constraints,^114^ which enforced a minimal flux through the microbial reactions at a fixed ratio of 400 times the flux through the biomass reaction. A consequence of these biomass reaction couplings is that if a microbial species produces any metabolite in the pathway of the predicted metabolites in blood, a non-zero shadow price is found. Therefore, the shadow prices of the microbial biomass metabolites reveal which microbial species can contribute to the predicted fluxes. These shadow prices were automatically computed when performing FBA and were extracted from *fba.y* after performing *fba = optimizeWBModel(model)*. Potential microbial influencers were then identified for each FBA prediction by assessing whether the shadow price value was zero, meaning that a change in microbial biomass would not change the flux prediction, or non-zero, indicating that altered microbial abundances could influence the flux results. A numerical tolerance for the shadow prices of 1e-6 was used to remove non-zero shadow prices resulting from numerical imprecision. After extracting the microbial species with non-zero shadow prices in each FBA prediction, microbial species with an average non-zero biomass shadow price for their associated flux predictions across all analysed samples were identified by calculating the bootstrapped average and associated 95% confidence interval for the microbial biomass shadow price across the analysed samples and each metabolic blood flux prediction. Bootstrapping was performed with 10,000 samples with replacement. Microbial species with cohort-average shadow prices that did not include zero in their 95% confidence intervals were flagged as potential contributors to the altered flux predictions in AD patients. Taken together, potential microbial influencers of the flux predictions were identified by analyzing the flux-associated shadow prices of the pan-biomass metabolites of microbial species included in the microbiome-personalized WBMs.

#### 4.13.2. Selection of flux-associated microbes

After identifying the microbial species that could have contributed to the flux predictions, we selected, for each AD-associated metabolic prediction, a minimal subset of flux-associated microbial species whose relative abundances best predicted the flux values. These sets of flux-associated microbes were identified via LASSO regressions. LASSO is an extension of ordinary least squares regression, which aims to minimize the number of non-zero coefficients, i.e., minimization of the L1 norm, for the predictor variables in addition to the sum of squared residuals, as in ordinary least squares regression. The inclusion of this minimization term enables the identification of the smallest set of covariates that predict the response variable with the lowest sum of squared residuals. The LASSO regression models were defined by: $\mathrm{Flux}∼\beta_{1}R_{1}+\beta_{2}R_{2}\ldots +\beta_{n}R_{n}+Sex$ . The predictors $R_{1}–R_{n}$ represent the relative abundances of the microbial species that were identified as potential influencers of the predicted blood fluxes in the shadow price analysis. The sex variable was added to account for differential fluxes between male and female WBMs. No other confounders were included, as gut microbial relative abundances and host sex were the sole sources of variation within the flux predictions. All LASSO regressions were performed using the lasso.m MATLAB function on z-transformed flux values and z-transformed microbial species relative abundances. A key limitation of LASSO regression is that the vector of regression coefficients is not unique, meaning that different sets of microbial species may satisfy the optimal regression fit. To ensure a robust and reproducible selection of microbial species, we utilized the stability selection method,^35^ which avoids the instability of covariate selection by performing LASSO on a large number of bootstrapped subcohorts and selecting only those covariates with a sufficiently high selection frequency. For this study, 500 random subcohorts were generated, each containing 70% of the analysed samples. A minimal selection frequency of 90% was chosen for the selection cut-off. To summarize, the stability selection method was used to identify minimal sets of microbial species that best explained the AD-associated blood fluxes.

#### 4.13.3. Calculation of microbe importances for the predicted blood fluxes

After identifying the minimal sets of microbial species for each predicted AD-associated blood metabolite, the prediction accuracy and explained variance of these microbial sets were predicted by performing elastic net regressions^37^ as follows: $\mathrm{Flux}∼\beta_{1}R_{1}+\beta_{2}R_{2}\ldots +\beta_{n}R_{n}+Sex$ , again with z-transformed flux values and relative abundances. Elastic net regressions were chosen as they are well-suited to handle a higher number of predictors and multicollinearity, similarly to LASSO regressions, but produce more stable regression coefficients. Elastic net regressions extend the LASSO paradigm with a RIDGE term, which minimizes the Euclidean norm, or L2-norm, of the predictor coefficients. Thus, elastic net regression aims to fit a regression line by minimizing (i) the sum of squared residuals, (ii) the number of non-zero coefficients (LASSO term), and (iii) the magnitude of the non-zero coefficients (RIDGE term). The LASSO and RIDGE terms are weighted by a parameter λ=0.5 , meaning that the importance of the LASSO and RIDGE terms is half the importance of minimizing the sum of squared residuals. The 95% confidence intervals and associated *P*-values for the predicted microbial elastic net regression coefficients were estimated by generating probability distributions for elastic net regression coefficients. These probability distributions were obtained by generating 100,000 random bootstrap samples with replacement and obtaining the elastic net regression coefficients for the microbial predictors in each bootstrap sample. The corresponding *P*-values were estimated by taking two times the minimal value of (a) the proportion of coefficients in the bootstrap probability distribution that were larger than zero, and (b) the proportion of coefficients that were below zero. This operation is analogous to a two-tailed t-test.

### 4.14. Software

Modeling work, including the construction and analysis of the microbiome WBMs, was done in MATLAB 2020b and MATLAB 2024b (Mathworks^TM^). In addition, the Parallel Computing Toolbox, Statistics and Machine Learning Toolbox, and the Bioinformatics Toolbox were utilized to generate and analyze microbiome WBMs. The generation and analysis of the microbiome-personalized WBMs were performed using functions from the COBRA toolbox v3,^112^ including the microbiome modeling^113^ and PSCM^20^ toolboxes. The IBM ILOG CPLEX 12.10 linear solver (IBM, Inc.) was used for performing FBA on the investigated metabolites. Visualization was performed in MATLAB and R version 4.5.0 using the tidyverse family of packages^118^ and the pheatmap package.^119^

## Supplementary Material

Supplementary tables Hensen et al for resubmission to Gut Microbes.xlsx Supplementary tables Hensen et al for resubmission to Gut Microbes.xlsx

## Data Availability

Samples were provided to NCRAD by ten Alzheimer’s Disease Research Centres, including UC San Diego, UC Davis, Stanford University, Kansas University, Wisconsin University, Indiana University, New York University, Wake Forest University, Cleveland Clinic, Nevada, and the University of Alabama at Birmingham. Clinical data can be requested from the National Alzheimer’s Coordinating Centre (naccdata.org/). Data will be available in the Synapse AD Knowledge Portal. The gut microbiome data are stored and accessible via the University of California, San Diego Qiita platform (qiita.ucsd.edu/). The AGORA2 reconstructions can be freely downloaded from the VMH resource (https://www.vmh.life/files/reconstructions/AGORA2/), while the APOLLO reconstructions are available from the Harvard Dataverse (version 1.0 https://doi.org/10.7910/DVN/PIZCBI).

## Code availability statement

All scripts can be accessed at https://github.com/ThieleLab/CodeBase.
